# Supplementary material for: Navigating persuasive strategies in online health misinformation: An interview study with older adults on misinformation management
Source: PLoS One. 2024 Jul 25;19(7):e0307771. doi: 10.1371/journal.pone.0307771 (PMC11271879; doi:10.1371/journal.pone.0307771)
Supplement: S1 Appendix — (DOCX) [file pone.0307771.s001.docx]

**Appendix 1: Four health misinformation articles with annotated persuasive strategies**

**Misinformation article 1: Cashews can treat depression**

Two Handfuls of Cashews is the Therapeutic Equivalent of a Prescription Dose of Prozac

Two handfuls of cashews each day may keep depression at bay. A growing body of research has found that in lieu of taking a prescription drug, some people can turn to foods that are high in tryptophans, like cashews.

Depressive episodes are often triggered when the body drops in serotonin and tryptophans can boost it again. One natural source of tryptophan is cashews. “Several handfuls of cashews provide 1,000-2,000 milligrams of tryptophan, which will work as well as prescription antidepressants,” says Dr. Andrew Saul, a therapeutic nutritionist and editor-in-chief of Orthomolecular Medicine News Service. The body turns tryptophan into serotonin, a major contributor to feelings of sexual desire, good mood, and healthy sleep.

What makes cashews superior to antidepressants is that you will surely avoid side effects that are usually caused by the medicine. Of course, this is yet another secret that big pharma will NEVER admit … there is much more effective and safer natural depression treatment they want to hide from the common people so they can continue to steal their money.

Also, cashews are delicious and can be found in milk or butter form. It is also possible to take cashews and turn them into something one can eat every day to keep depression at bay.

**Misinformation article 2: Radiation contaminated pet food**

BEWARE that the food you purchase for your fur friends can kill them! The government has made deals with Japan, China and Korea to import seafood from the waters near Fukushima for pet food manufacture. My friend has a Geiger counter inherited from her dad who was a nuclear physics professor. Putting it within 1 inch of the cat wet food, the reading exceeded the limit by three fold. This is terrifying! She sent me a chart showing the wave height of tsunami. This is also the radioactive discharge emanating from Fukushima ever since. Now Japan has confirmed that it will start releasing contaminated water from the Fukushima Daiichi nuclear power stations into the Pacific within two years. The whole pacific ocean will be contaminated!

**https://www.snopes.com/fact-check/fukushima-emergency/**

**Misinformation article 3: Masking not necessary and COVID is just flu**


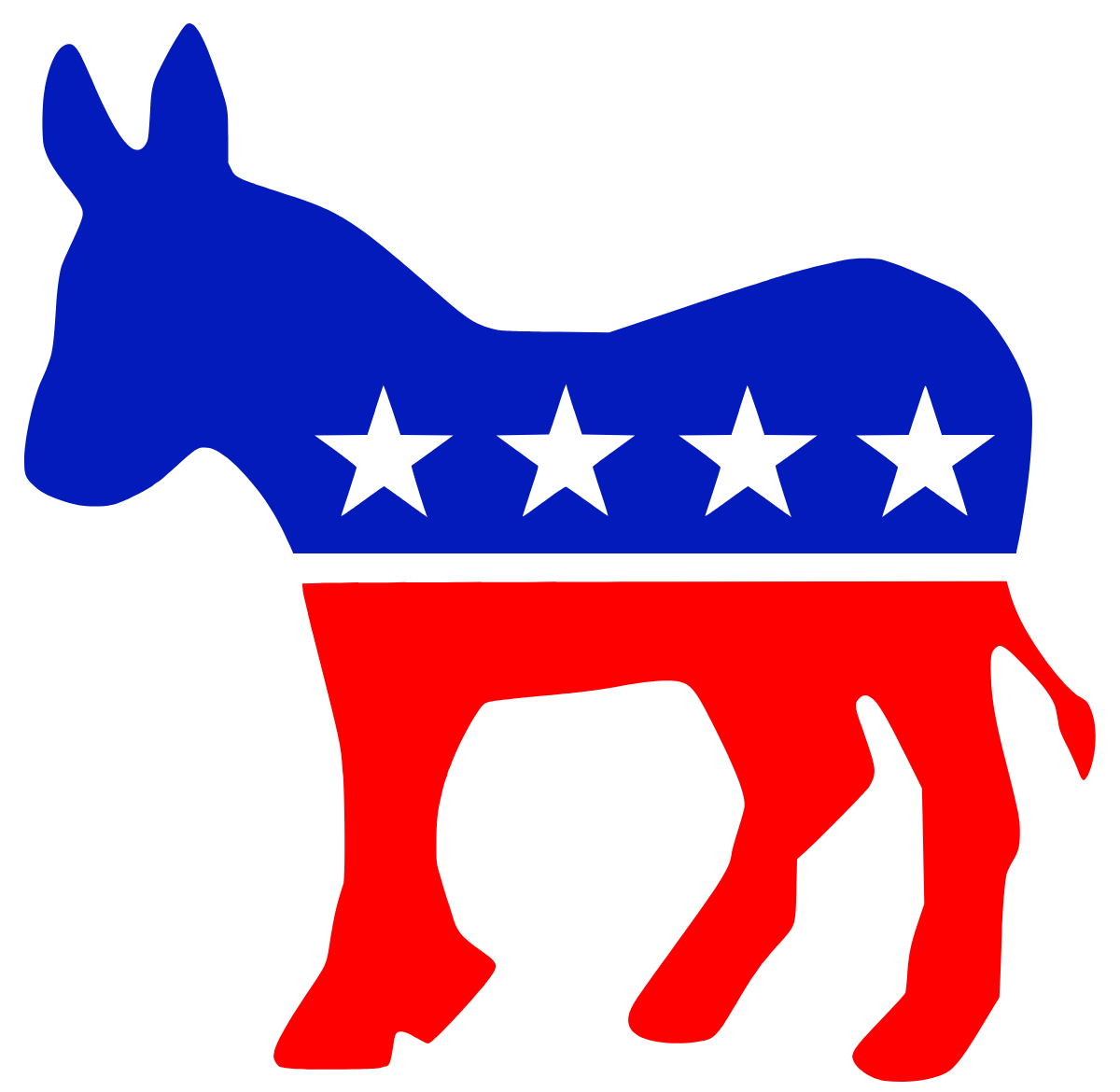
Gov. Jim Wik just passed the mask mandate! Covid is just like flu: survival rate among 50 Y/O and under is >99.9%. This mandate also prohibits churches from holding indoor services without wearing masks. Forcing you to put on a mask is trying to take away your freedom and control you!

**Misinformation article 4: Sunscreen causes cancer**

As some of you may already know, I was diagnosed with stage 1 breast cancer last year. My family has no history of cancer so I kept on wondering why me? As I continued with my treatment, I came across an article about sunscreen. I have used sunscreen all my life because I was told by professionals that it would keep me safe from the sun. When I finally did my research, I discovered that the skincare industry has been LYING to us this entire time. They told us sunscreen can protect us from skin cancer, but what they hid from us is that sunscreen contains ingredients like paraben and oxybenzone, which are carcinogens. The only thing they care about is MONEY, so they lied to us - that is ENRAGING.

Paraben is a preservative used in many sunscreen brands and can be easily absorbed into our bloodstreams through the biggest organ on the human body, our skin. These companies cite research arguing a small amount won’t cause any harm to us but what about the accumulation of these toxic chemicals inside our bodies over time? We all know scientific data are delayed and who knows when they may release new data one day retracting the previous findings.

Link to article: https://www.huffpost.com/entry/trans-fats-of-the-skin-ca_b_112236

Comments:

David: Thanks for the info, Molly! Are there any research articles to support this?

Molly’s reply: You’re very welcome! From what I have seen, there is minimal research done on the long-term effects of sunscreen use.
