# Supplementary material for: Navigating persuasive strategies in online health misinformation: An interview study with older adults on misinformation management
Source: PLoS One. 2024 Jul 25;19(7):e0307771. doi: 10.1371/journal.pone.0307771 (PMC11271879; doi:10.1371/journal.pone.0307771)
Supplement: S2 Appendix — (PDF) [file pone.0307771.s002.pdf]

Pinterest post

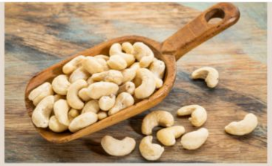

TWO HANDFULS OF CASHEWS IS THE THERAPEUTIC EQUIVALENT OF A PRESCRIPTION DOSE OF PROZAC

WWW.WELLHEALTH.COM

Two handfuls of cashews each day may keep depression at bay. A growing body of research has found that in lieu of taking a prescription drug, some people can turn to foods that are high in tryptophans, like cashews.

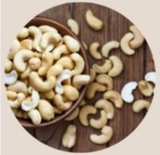

Depressive episodes are often triggered when the body drops in serotonin and tryptophans can boost it again. One natural source of tryptophan is cashews. “Several handfuls of cashews provide 1,000-2,000 milligrams of tryptophan, which will work as well as prescription antidepressants,” says Dr. Andrew Saul, a therapeutic nutritionist and editor-in-chief of Orthomolecular Medicine News Service. The body turns tryptophan into serotonin, a major contributor to feelings of sexual desire, good mood, and healthy sleep.

What makes cashews superior to antidepressants is that you will surely avoid side effects that are usually caused by the medicine. Of course, this is yet another secret that big pharma will NEVER admit ... there is much more effective and safer natural depression treatment they want to hide from the common people so they can continue to steal their money.

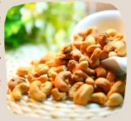

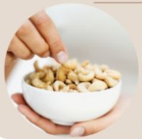

This is great news for people who want a more natural response to their depression or for those who react terribly to Prozac. Also, cashews are delicious and can be found in milk or butter form. It is also possible to take cashews and turn them into something one can eat every day to keep depression at bay.

Save

Two Handfuls of Cashews is the Therapeutic Equivalent of a Prescription Dose of Prozac

2 handfuls of #cashews is the therapeutic equivalent of a prescription dose of prozac! #benefits #health #healthylife

HWell Health

Photos Comments

Tried this Pin?
Add a photo to show how it went

Add photo

Reddit post

r/AlzheimersGroup · Posted by [illegible] 3 days ago

Benefits of Caffeine for Alzheimer's disease

Caffeine, contained in coffee, has a central stimulating effect, like other purine bases, and is said to have many positive and negative somatic characteristics. There are longitudinal studies that identify a 16% reduced risk of being affected by Alzheimer's disease among coffee drinkers. However, it is still uncertain if caffeine alone is responsible for this reduced risk or if other ingredients and factors are also relevant. Coffee can therefore only possibly reduce the risk of Alzheimer's disease.

Award Share Save Hide Report

Instagram post

[redacted]
Rochester, Minnesota

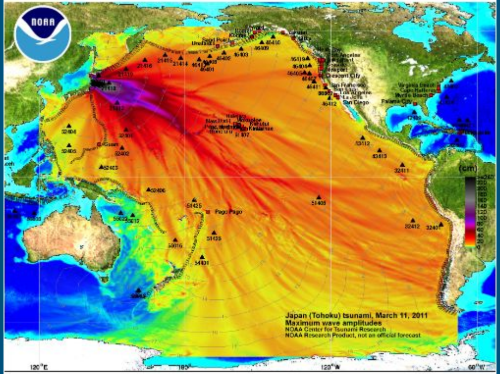

[redacted]

[redacted] BEWARE that the food you purchase for your fur friends can kill them! The government has made deals with Japan, China and Korea to import seafood from the waters near Fukushima for pet food manufacture. My friend has a Geiger counter inherited from her dad who was a nuclear physics professor. Putting it within 1 inch of the cat wet food, the reading exceeded the limit by three fold. This is terrifying! She sent me a chart showing the wave height of tsunami. This is also the radioactive discharge emanating from Fukushima ever since. Now Japan has confirmed that it will start releasing contaminated water from the Fukushima Daiichi nuclear power stations into the Pacific within two years. The whole pacific ocean will be contaminated!
#Pets #Animals #Petlovers #Petstagram #Nature #Responsiblepetowner #Protectnanimals ... more

[redacted]

JULY 31

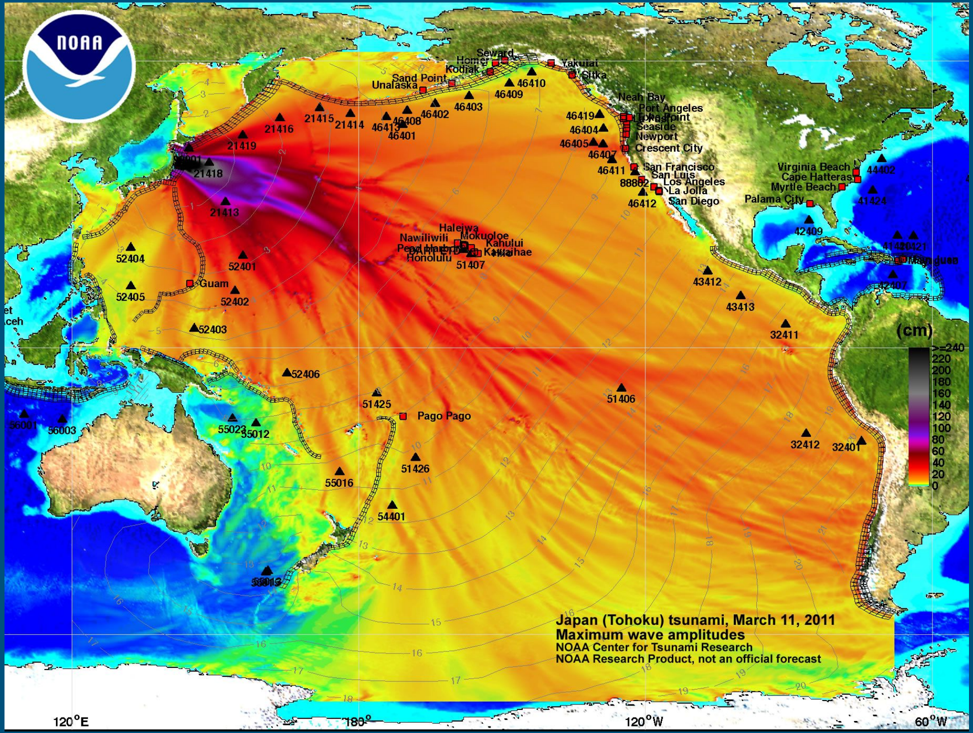

[redacted]

Dem Gov. [redacted] just passed the mask mandate! Covid is just like the flu: survival rate among 50 Y/O & under is >99.9%. This mandate also prohibits churches from holding indoor services without wearing masks. Forcing you to put on a mask is trying to take away your freedom and control you!

12:23 PM · Jul 17, 2021

[redacted]

Twitter Post/Tweet

Facebook Post

[redacted]

August 19 at 9:45pm ·

As some of you may already know, I was diagnosed with stage 1 breast cancer last year. My family has no history of cancer so I kept on wondering why me? As I continued with my treatment, I came across an article about sunscreen. I have used sunscreen all my life because I was told by professionals that it would keep me safe from the sun. When I finally did my research, I discovered that the skincare industry has been LYING to us this entire time. They told us sunscreen can protect us from skin cancer, but what they hid from us is that sunscreen contains ingredients like paraben and oxybenzone, which are carcinogens. The only thing they care about is MONEY, so they lied to us - that is ENRAGING.

Paraben is a preservative used in many sunscreen brands and can be easily absorbed into our bloodstreams through the biggest organ on the human body, our skin. These companies cite research arguing a small amount won't cause any harm to us but what about the accumulation of these toxic chemicals inside our bodies over time? We all know scientific data are delayed and who knows when they may release new data one day retracting the previous findings.

Link to article: https://www.huffpost.com/entry/trans-fats-of-the-skin-ca_b_112236

[redacted] [redacted]

[redacted] Comment Share

[redacted]

[redacted] Thanks for the info, [redacted] Are there any research articles to support this? [redacted]

Like · Reply · 3w

[redacted] You're very welcome! From what I have seen, there is minimal research done on the long-term effects of sunscreen use. [redacted]

Like · Reply · 3w

Write a comment...

Online Article

HealthyNews Health Conditions Discover Plan Connect Subscribe

HEALTH NEWS

Written by Shawn Hart on September 4, 2021

Long-term effectiveness of covid vaccine

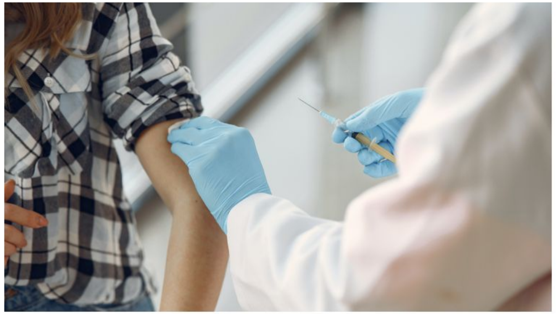

Photo by Natalie Johnson

If you are fully vaccinated, you may be excited about gathering with family and friends again. However, you may be displeased to hear that public health officials are unsure about how long the protection from the coronavirus vaccines will last. For instance, will your shot wear off gradually or suddenly? Will you need a booster?

Pfizer and Moderna continue to monitor immunity in people who were given their vaccines in the initial clinical trials—both companies reported strong overall efficacy at the six-month mark. A report in The New England Journal of Medicine (NEJM) showed that 33 participants who had received the Moderna vaccine during the Phase I trial had a gradual decline in antibody protection—and, based on the slope, Akiko Iwasaki, a professor of immunobiology at Yale School of Medicine, says, “The slow decline raises hopes that the mRNA vaccines will be protective for at least a year.”

Other experts hold similar optimistic views. Immunologist Jennifer Gommerman at the University of Toronto said that, although about six months after the shots, the antibodies in the blood have fallen...but in vaccinated people, these infections will most likely be mild or moderate because the immune system isn't starting from scratch. In fact, it's the opposite. It has been training cells and antibodies for months.

However, Dr. Meyer added, “The length of immunity is somewhat dependent on the patient.” While more research is needed, there could be variations in immune responses from person to person based on such factors as age, medical conditions, and medications they may be taking.

Exactly how long the protection from mRNA vaccines will last is uncertain. In the absence of variants that sidestep immunity, in theory immunity could last for years, experts said. But the virus is clearly evolving. So until more is known, don’t rely solely on the vaccine.

FEEDBACK:

If you are fully vaccinated, you may be excited about gathering with family and friends again. However, you may be displeased to hear that public health officials are unsure about how long the protection from the coronavirus vaccines will last. For instance, will your shot wear off gradually or suddenly? Will you need a booster?

Pfizer and Moderna continue to monitor immunity in people who were given their vaccines in the initial clinical trials—both companies reported strong overall efficacy at the six-month mark. A report in The New England Journal of Medicine (NEJM) showed that 33 participants who had received the Moderna vaccine during the Phase I trial had a gradual decline in antibody protection—and, based on the slope, Akiko Iwasaki, a professor of immunobiology at Yale School of Medicine, says, “The slow decline raises hopes that the mRNA vaccines will be protective for at least a year.”

Other experts hold similar optimistic views. Immunologist Jennifer Gommerman at the University of Toronto said that, although about six months after the shots, the antibodies in the blood have fallen...but in vaccinated people, these infections will most likely be mild or moderate because the immune system isn't starting from scratch. In fact, it's the opposite. It has been training cells and antibodies for months.

However, Dr. Meyer added, “The length of immunity is somewhat dependent on the patient.” While more research is needed, there could be variations in immune responses from person to person based on such factors as age, medical conditions, and medications they may be taking.

Exactly how long the protection from mRNA vaccines will last is uncertain. In the absence of variants that sidestep immunity, in theory immunity could last for years, experts said. But the virus is clearly evolving. So until more is known, don’t rely solely on the vaccine.

FEEDBACK:
