# Supplementary material for: Navigating persuasive strategies in online health misinformation: An interview study with older adults on misinformation management
Source: PLoS One. 2024 Jul 25;19(7):e0307771. doi: 10.1371/journal.pone.0307771 (PMC11271879; doi:10.1371/journal.pone.0307771)
Supplement: S3 Appendix — (DOCX) [file pone.0307771.s003.docx]

Appendix 3: Interview guide

1. **General perception of misinformation**
2. Have you ever encountered “misinformation”? If so, what is the misinformation?

Probe: Health misinformation?

1. *Where* did you encounter the misinformation?
2. Can you tell me about *your experience* with misinformation?

Probe: Positive/ negative experiences with misinformation in daily experiences. Can you give me an example?

1. How do you *define misinformation*? In other words, what do you think “misinformation” is?

(if it’s difficult for the participant to come up with a definition, we can say “for instance, *what features in the information* help to tell whether it’s true or misinformation?”

1. How do you *identify* misinformation? Any signature/feature/red flag for misinformation?
2. When you encounter such misinformation, *how do you manage misinformation*? [probe: how do you act on the misinformation? Dismiss? Join the discussion?]
3. Do you have any *difficulties* in handling misinformation? e.g., Do you feel you can tell whether it’s misinformation?
4. What can be the situation where you (or others) need most support in terms of managing health-related misinformation?
5. **Show the trigger material (6 messages)**

Now I am going to show you the six health information collected online via my shared screen. Some are misinformation and some are not. [**Share Screen Now with the trigger material**. We define online health misinformation as “health-related information disseminated on the Internet that is false, inaccurate, misleading, biased, or incomplete, which is contrary to the consensus of the scientific community based on the best available evidence.”

Most of the information is from social media but we masked information such as number of likes, the specific names, and profile pictures so that those will not impact your judgment of the content of the information. For each piece, please read the information and then I will ask you a few questions.

We have found that we get a better understanding if we ask people to *think aloud as they read the informatio*n. All you have to do is speak your thoughts as you read along. It may be a bit awkward at first, but it gets easier once you get used to it. Basically, we’d like any thought that comes into your head to come out your mouth while reading the health information. After you read each piece of information and *verbalize your thoughts*, For each message, we will ask you a bunch of questions after you read it.

**Note:** If the participant becomes quiet or just reads out the text but does not verbalize their thoughts, say: “I’d like to hear what you're thinking; If you could just say whatever words come to your mind; or Please speak up”.

1. **Perception of misinformation persuasive strategies (5 questions)**
2. Have you read through the whole text?
3. What do you think is the main argument of this article?
4. Do you agree or disagree with it, or partially agree, partially disagree? Why or why not?

Probe:

1. What about this information made you think so (disagree/agree/partially agree and partially disagree)?
2. Are there any particular parts of the information that made you think so/suspicious?
3. What cues, evidence, or heuristics do you use to make the judgment?
4. **Have you heard about similar information in the example before?**

Probe:

1. Can you give me an example?
2. From personal experience, families, friends
3. Having experience related to the information?
4. **Do you think people would think this information is believable? Why or why not?**

Probe:

1. What specific elements of the information made you think that it is believable/it could be believable/it is not believable?
2. Which statements in this example made strong impressions on you? Why?
3. **What actions do you think you would take after reading this article/post?**

Probe:

1. Personal actions such as behavior change?
2. Tell other people about this?
3. Look for more information about this issue?
4. Discuss with others who you trust?

7. Is there anything else you would like to add about this piece of information you just read?

**Closing**
