# Supplementary material for: Navigating persuasive strategies in online health misinformation: An interview study with older adults on misinformation management
Source: PLoS One. 2024 Jul 25;19(7):e0307771. doi: 10.1371/journal.pone.0307771 (PMC11271879; doi:10.1371/journal.pone.0307771)
Supplement: Attachment — Submitted filename: Response to Reviewers .docx [file pone.0307771.s004.docx]

Dear Editor

In this letter, we provided point-to-point responses addressing each of the issues raised. We would like to thank the reviewers for providing us constructive feedback, which helped us revise and improve our manuscript. We hope the reviewers will find our revised manuscript satisfactory this round. Below, we first respond to the editor’s comments and then the reviewers’ comments.

**Responses to Editor’s comments:**

1. We formatted the manuscript and the title page according to PLOS ONE's style requirements, [https://journals.plos.org/plosone/s/file?id=wjVg/PLOSOne_formatting_sample_main_body.pdf](https://urldefense.com/v3/__https:/journals.plos.org/plosone/s/file?id=wjVg*PLOSOne_formatting_sample_main_body.pdf__;Lw!!HXCxUKc!zjpEphURHleQptdjBJubEiPoZz736ZamhN_tcxJkT_tpqynz-ChlwZEpKS8p9KEwXd3E3_2r6Wt-0NhH4w$) and
    <https://journals.plos.org/plosone/s/file?id=ba62/PLOSOne_formatting_sample_title_authors_affiliations.pdf>. Together with this rebuttal letter (response to reviewers), we also submitted a marked-up copy of our manuscript (Revised Manuscript with Track Changes) that highlights changes made to the original version, and an unmarked version of your revised paper without tracked changes (Manuscript).
2. “In the ethics statement in the Methods, you have specified that verbal consent was obtained. Please provide additional details regarding how this consent was documented and witnessed, and state whether this was approved by the IRB.”

Response: We added the details of the consent process in the Methods–data collection and procedure section.

3. Thank you for stating the following financial disclosure:
    [Brandt Fellowship].
    Please state what role the funders took in the study. If the funders had no role, please state: ""The funders had no role in study design, data collection and analysis, decision to publish, or preparation of the manuscript.""
    If this statement is not correct you must amend it as needed.
    Please include this amended Role of Funder statement in your cover letter; we will change the online submission form on your behalf.

Response: We have added the statement regarding the funder’s role in the cover letter.

4. We note that you have indicated that there are restrictions to data sharing for this study. PLOS only allows data to be available upon request if there are legal or ethical restrictions on sharing data publicly. For more information on unacceptable data access restrictions, please see [http://journals.plos.org/plosone/s/data-availability#loc-unacceptable-data-access-restrictions](https://urldefense.com/v3/__http:/journals.plos.org/plosone/s/data-availability*loc-unacceptable-data-access-restrictions__;Iw!!HXCxUKc!zjpEphURHleQptdjBJubEiPoZz736ZamhN_tcxJkT_tpqynz-ChlwZEpKS8p9KEwXd3E3_2r6WvP_3N-tQ$).

Response: Our consent form stated: “**PRIVACY AND CONFIDENTIALITY:** Your confidentiality will be protected to the maximum extent allowable by law. Specifically, your interaction will be audio recorded, and we will collect outcomes (e.g., photos). We will use zoom and Microsoft to transcribe the audio. Once the transcription is complete, we will delete the audio recording. The online questionnaire is anonymous. The deidentified transcriptions will be kept confidential and private in university approved servers and computers that are password protected. The data will be stored for three years. Your contact information will be saved for us to send you the participation incentive or contact you back for the next phase of this study. Otherwise, all information will be destroyed once de-identification has been completed. The data collected from this study will only be used within our research team and will not be distributed to others.”. We promised the participants that the deidentified data, i.e., transcripts will only be used within our research team and will not be distributed to others. Therefore, we cannot share the data. The study is approved by Michigan State University’s Institutional Review Board (Study ID: STUDY00006370). Michigan State University’s Human Research Protection Program at 517-355-2180, Fax 517-432-4503, or e-mail [irb@msu.edu](mailto:irb@msu.edu) or regular mail at 4000 Collins Rd, Suite 136, Lansing, MI 48910.

5. Please include your full ethics statement in the ‘Methods’ section of your manuscript file. In your statement, please include the full name of the IRB or ethics committee who approved or waived your study, as well as whether or not you obtained informed written or verbal consent. If consent was waived for your study, please include this information in your statement as well.

Response: We have added the details at the beginning of the Methods–Data collection and procedure section.

**Responses to reviewers’ comments:**

Reviewer #1:

Thank you for the opportunity to read the article entitled ‘Navigating persuasive strategies in online health information: An interview study with older adults’. The manuscript describes a qualitative interview study about how older adults identify and cope with online health-related misinformation. The manuscript addresses an important topic from a qualitative angle, and provides rich data and potentially in-depth insights in how older people attend to and cope with online misinformation. It is clear from the manuscript that the authors have put a lot of effort in the data collection, data analysis and report of the study. I find the topic interesting and important and believe that the richness of the qualitative data can add something to the literature on online health-related misinformation attention, awareness and effects. At the same time I have some questions and concerns which I will mention point by point below, and I hope that they are helpful to the authors in the strengthening of their manuscript.

**Response**: Thank you very much for your constructive feedback!

Major points
 - There is a lot of information in this manuscript – which is common for a qualitative study. Therefore, a clear structure is vital to guide the reader through the manuscript and understand the focus of the study. The most easy way to do this - in my view is - to formulate clear aims and research questions and use those to clearly structure the manuscript. The current manuscript formulates research questions and aims, but their relation with how the rest of the manuscript is structured (e.g., the theory section and results section) was not always clear to me. As a result I felt often lost while reading as so many topics are discussed.

**Response**: Thank you for the comment. We now clearly stated the research questions and revised the literature review and result section to structure them in accordance with the research questions.

- The title mentions ‘persuasive strategies’ which is a central theme to the study. These strategies have been identified in a prior literature review conducted by the authors. I find this classification interesting, and unfortunately due to anonymity I cannot check the article they refer to. The twelve strategies are central to the first part of the literature review. In my view, the 12 strategies would better fit the method section (e.g., in the form of a text box) as they are clearly part of the operationalization of the study. I assume that the strategies have been used to develop the messages that the participants were presented with, or that at least that the strategies can be identified in the messages. The literature review, I would expect to discuss more board themes/categories of misinformation characteristics (in contrast to the individual strategies that were identified in a single study). For example in terms of layout/format, content, role of scientific evidence, to place the strategies in a broader context of the literature.

**Response:** The 12 persuasive strategies indeed were the foundations to develop the messages they participants were exposed to. The literature review section did include the board description and we now add an annotation of the 12 persuasion strategies in the four misinformation messages (appendix 1) and the six pieces of messages the participants were exposed to (appendix 2). We also realized that PLOS ONE uses single-blind review, not double-blind review. Therefore, we added the citations of our own paper.

- The literature review could perhaps focus more on older adults in particular and what is known about this group in terms of encountering, attending, and responding to (e.g., reasons to check or share) health-related misinformation online and a discussion of the distinction between misinformation (can be false information, but unintendedly or being outdated) and disinformation (intendedly false information).

**Response**: Because this study particularly focused on people, especially older adults’ responses to persuasive strategies in online health misinformation as well as their management tactics, we wrote the literature review to focus particularly on these areas and did not stretch to broader literature related to encountering, attending, responding and sharing of misinformation. Nevertheless, we agree with the reviewer’s point about reviewing literature specifically focused on older adults. We expanded our current literature review to add a few studies specific to older adults in the domain of management of misinformation, especially related to attending and responding to misinformation. Please see the revised manuscript with the track change.

We also added clarification of this definition in relation to disinformation right after we introduce the definition of “online health misinformation” at the beginning of the manuscript.

- I was surprised that some theoretical mechanisms that are important to misinformation effects, such as confirmation bias, are only mentioned in the results section and not in the literature review section (as a reason why people believe misinformation).

**Response:** Thank you for the comment. The literature review section discussed “compatibility with prior belief”and did not specifically use the term confirmation bias. We updated the literature to make the concept of confirmation bias more specific.

- At some points, some rather strong conclusions are drawn upon this qualitative study and I think that the authors could be a bit more careful in their formulation. For example the conclusion on page 21 mentions that “based on the findings media literacy is a solution to mitigate misinformation susceptibility”. As media literacy is not measured or assessed in some way in this study, this should perhaps be formulated more carefully.

**Response:** Thank you for the comment. The concept of media literacy was not reviewed or discussed earlier and was abruptly introduced and in a manner as a conclusion on page. Nowe we have revised the text by adding a sentence to add the rationale for why media literacy education is needed.

*“Our findings have shown evidence of older adults falling for the common persuasive strategies while processing online health misinformation. To mitigate their susceptibility to the common persuasive strategies, older adults should be equipped with the knowledge of what they are and be able to identify the common tricks and pitfalls.”*

Another example is the statement on page 208 (misinformation usually incorporates the trope of big pharma being evil). A reference would be helpful here. I also wonder whether this statement refers to misinformation or mainly disinformation.

**Response:** Indeed, we were referring to one of the persuasive strategies discussed in the literature review. As this is the result section, we did not have a reference. However, we understand the reviewer’s concern. We now have removed this sentence to focus on the results since this persuasive strategy was already discussed in the literature review.

- Some critical information about the study is missing in the methods section. These are for example, information about how and where the participants of this study were recruited, when participants qualified as ‘older adults’ – what was the minimum age? Did other inclusion/exclusion criteria apply? In which country was the study conducted? I assume it was in the US but would be good to mention. Who conducted the interviews?

**Response**: Detailed information of how and where the participants were recruited, inclusion criteria are now added.

- It would be informative to see the articles that were discussed with the participants added as appendix to the manuscript. This would allow the reader to see how the various strategies were incorporated in each article. Am I correct that all 12 persuasive strategies were incorporated in each article? How was this done? What was the length of the articles? What role played images (if they were there)? Where they all designed in the form of a Facebook post? (line 203).

Response: Please see appendix 2 of the content of these messages. The messages were designed to be posts of various popular online platforms, including a pinterest post, a Reddit post, a tweet, a Facebook post, an instagram post, and a website article. Please see appendix 1 of the annotation of the persuasive strategies in the four pieces of misinformation.

- I also wonder whether the authors tested the knowledge of the participants on the topics of the messages prior to the study. I can imagine that peoples prior knowledge might also (or mainly) impact people’s ability to sport misinformation, perhaps more than the persuasive strategies within the articles.

**Response**: We did not test their prior knowledge using survey instruments. However, in the interview, we asked them whether they have heard of this information or similar information before. Except for the COVID-19 related messages, participants mostly indicated that they had little prior knowledge of the topics of the message. We also added one sentence regarding prior knowledge to provide contextual information in the revised manuscript.

- There is no limitation section in the article.

**Response**: Thank you for the comment. We added the limitation section.

Minor points
 - Throughout the manuscript, it says “12 groups of persuasive strategies”. This is a bit confusing, because 12 is already a lot and the word groups implies that each strategy consists of multiple strategies. In the discussion section on page on page 12 (line 475) the word group is left out, which I find more logical and clear.

**Response**: It is 12 groups of persuasive strategies because some groups have sub-group strategies. We consistently used 12 groups of persuasion strategies now.

- It is mentioned that the interviews were part of a larger study and that other parts of the findings are reported elsewhere. For reasons of transparency I would advise the authors to provide references to related studies here.

**Response**: This article is the first article submitted for journal publication, although we do have a conference paper focusing on different research questions. We want to be clear that although the data came from the same dataset but completely different research questions were examined. We now added the citation of the conference paper. The conference paper is in the process of being submitted to another journal.

- The example questions from the interview guide mentioned on page 7/8 are helpful. The authors might consider adding the entire interview guide as an appendix.

**Response**: We added the complete interview guide in appendix 3.

- Who did the coding of the interviews? Did the first and second coder code the interviews independently?

**Response**: Four people were involved in coding, including the first two authors (communication professors) and two individuals (graduate students) who were not co-authors. Each transcript was coded independently by two coders. We added the details in the data analysis section.

Reviewer #2: Thanks for the opportunity to review this manuscript which delves into the perspective of older adults while navigating persuasive online health misinformation. The attention on the specific population subgroup is needed, and the methodological approach is adequate. I believe the manuscript could be improved by addressing the comments listed below.

**Response**: Thank you for your constructive feedback!

Title: I would encourage the authors to try finding a more informative title.
**Response**: We revised the title to: Navigating persuasive strategies in online health misinformation: An interview study with older adults on misinformation management

Abstract Revision:
It would be beneficial to consider explicitly stating the research gap that the study addresses at the beginning of the abstract, as this would provide readers with a clearer understanding of the study's significance.

**Response**: We have revised the beginning of the abstract by expliciting stating the gap.
“Online health misinformation commonly includes persuasive strategies that can easily trick lay people. Yet it is not well understood how individuals respond to misinformation with persuasive strategies at the moment of exposure. This study aims to address the research gap by exploring how and why older adults fall into the persuasive trap of online health misinformation and how they manage their encountering of online health misinformation.”

Methods Section:
Provide a more detailed explanation of the analysis process, including the coding procedures and criteria.
Clarify the reasons behind the reporting decision by stating that it was made to focus on the most relevant results while ensuring the paper's conciseness.
Include a table summarizing participant characteristics, and expand on population, sampling, and recruitment procedures.

**Response**: We have now added more details of the analysis process. We also added clarification that the reporting focuses on the most relevant results while ensuring the paper's conciseness. Details of the recruitment were added to the method section. Table 1 was added summarizing participant characteristics.

Empirical Study Design:
Offer more information about the design and selection of the misinformation used in the empirical study. Explain how this misinformation was chosen and its relevance to the research questions.
**Response**: Please see appendix 1 of the content of these messages. These messages were created based on the 12 groups of persuasive strategies. The messages were designed to be posts of various popular online platforms, including a pinterest post, a Reddit post, a tweet, a Facebook post, an instagram post, and a website article. Please see appendix 2 of the visual presentation.

Results Section:
Consider referencing more specific raw data or examples from the study to support the results and enhance their clarity.

**Response**: We added more quotes to illustrate the points, especially for the first research question. Please see the revised manuscript with the track change.

Discussion Section:
Add an introductory section to the discussion to provide context and set the stage for the subsequent points.

**Response**: An introductory section was added to the discussion on p. 23

Elaborate on how the findings relate to the idea of "improving media literacy," discussing its sustainability and feasibility in more depth.

**Response**: Elaborations on how findings related to “improving media literacy” are added on p. 25. Discussions about sustainability and feasibility are added in the new paragraph on p. 26.

Incorporate discussions about relevant dual-process theories like the Elaboration Likelihood Model (ELM) to better explain cognitive processing in the context of misinformation.

**Response**: Discussions about dual-process theories are incorporated in the new paragraph on p. 26.

Explore the significance of individuals being able to recognize their emotions, considering its implications for health misinformation discernment.

**Response**: Discussions have been strengthened on p. 27 concerning the implications of individuals being able to recognize their emotions.

Delve into the complexities and challenges associated with developing the competences necessary for discerning conflicting health evidence.

**Response**: Discussions are added about the complexities and challenges associated with developing the competences for discerning conflicting health evidence on pp.28-29.

Connect the influence of personal relationships as a moderator of misinformation's impact with the respective results to provide a clearer link.

**Response**: The connections are made clearer on p. 29 about the moderating role of personal relationships.

Minor Edits:
Follow the WHO naming conventions by using "COVID-19" instead of "Covid-19" throughout the paper.

**Response**: Thank you for pointing this out. We have changed throughout the paper.
