# Supplementary material for: Navigating persuasive strategies in online health misinformation: An interview study with older adults on misinformation management
Source: PLoS One. 2024 Jul 25;19(7):e0307771. doi: 10.1371/journal.pone.0307771 (PMC11271879; doi:10.1371/journal.pone.0307771)
Supplement: Attachment — Submitted filename: 2nd Response to reviewer.docx [file pone.0307771.s005.docx]

Reviewer #2: The manuscript in its present form has improved and most of the comments from reviewers have been adequately addressed.

However, I still believe that even if the introduction section is quite extensive, the reasons justifying the interest on the specific subpopulation (older adults) are not adequately presented. I would suggest dedicating a section to this and also reflecting specific considerations into the discussion.

I noticed a typo on page 2 line 60.

**Response**: We now have dedicated a section in the introduction to justify focusing on this specific subpopulation of older adults (the 3th paragraph of the Introduction).

We also added in the discussion section reflecting specific considerations of older adults (p. 28 and p. 30).

We also carefully proofread the manuscript for grammar and typos. Thank you!
